# Measuring the Senescence-Associated Secretory Phenotype

**DOI:** 10.3390/biomedicines13092062

**Published:** 2025-08-24

**Authors:** Achilleas Karras, Georgios Lioulios, Konstantia Kantartzi, Asimina Fylaktou, Stylianos Panagoutsos, Maria Stangou

**Affiliations:** 1 School of Medicine, Democritus University of Thrace, 68100 Alexandroupolis, Greece; achilleas199328@outlook.com (A.K.); kokan0910@gmail.com (K.K.); spanagou@med.duth.gr (S.P.); 2 Department of Nephrology, Papageorgiou Hospital, 56429 Thessaloniki, Greece; 3 School of Medicine, Aristotle University of Thessaloniki, 54642 Thessaloniki, Greece; pter43@yahoo.gr; 4 Department of Nephrology, University Hospital of Alexandroupolis, 68100 Alexandroupolis, Greece; 5 Department of Immunology, National Peripheral Histocompatibility Center, General Hospital Hippokration, 54642 Thessaloniki, Greece; fylaktoumina@gmail.com; 6 1st Department of Nephrology AUTH, Hippokration Hospital, 54642 Thessaloniki, Greece

**Keywords:** senescence-associated secretory phenotype, immunosenescence, quantitative reverse transcription, protein analysis, immunostaining

## Abstract

Cellular senescence is a fundamental hallmark of aging, contributing to tissue dysfunction and chronic disease through the senescence-associated secretory phenotype (SASP). The SASP encompasses a diverse and dynamic collection of secreted cytokines, chemokines, growth factors, and proteases that vary depending on cell type, senescence trigger, and microenvironmental context. Accurate quantification of SASP components is critical to understanding the mechanisms linking senescence to pathology and for advancing senotherapeutic strategies. However, measuring the SASP presents significant technical and biological challenges due to its complexity, heterogeneity, and context dependence. This review provides a comprehensive overview of the principal methodologies used to measure SASP components across different biological levels—transcriptional, translational, and functional—and sample types, including cell cultures, tissues, and systemic fluids. We discuss the advantages and limitations of widely used RNA-level techniques (e.g., qRT-PCR, RNA sequencing, in situ hybridization), protein-level assays (e.g., ELISA, Western blotting, mass spectrometry, Luminex, MSD), and spatial detection methods (e.g., immunohistochemistry, immunofluorescence). By organizing current SASP detection strategies by molecular level and sample source, this review highlights the importance of multiparametric approaches to capture the full spectrum of senescent cell activity. We also identify key methodological gaps and propose directions for refining SASP biomarker discovery in aging and disease research.

## 1. Introduction

Over the past century, global life expectancy has increased markedly due to improvements in healthcare, sanitation, and disease control. As a result, many countries are now experiencing unprecedented demographic shifts, with individuals aged 65 years and older comprising a rapidly growing segment of the population [1,2] While this trend reflects a public health success, it also presents major challenges: older age remains the greatest risk factor for a host of chronic conditions, including cardiovascular disease, diabetes, cancer, cognitive impairment, and functional decline [3,4]. These age-associated conditions significantly impact quality of life, healthcare systems, and economic productivity. Consequently, increasing healthspan, or the period of life spent in good health, has emerged as a primary objective in aging research [5].

The geroscience hypothesis provides a conceptual framework for achieving this goal. It proposes that by targeting the biological mechanisms of aging itself, rather than treating individual diseases in isolation, it may be possible to delay the onset of multiple age-related disorders simultaneously, thereby extending both lifespan and healthspan [6]. Among the established hallmarks of aging, cellular senescence has emerged as a key mechanistic contributor to age-related tissue dysfunction and chronic disease burden [7].

Senescence has been studied beyond the scope of common age-related conditions. There have been identified strong associations between specific SASP proteins and rare genetic diseases. For instance, in Fanconi anemia (FA), cells exhibit hallmark traits of senescence, such as activation of the p53–p21 pathway, altered telomere length, mitochondrial dysfunction, chromatin changes and a pro-inflammatory phenotype [8]. A landmark 2024 study shows increased cellular senescence markers in cystic fibrosis (CF) bronchial epithelium across human samples, cell lines, and cystic fibrosis transmembrane conductance regulator (CFTR) knockout rat models. This senescence is linked with upregulated SASP markers, fibroblast growth factor receptors (FGFRs) and mitogen-activated protein kinase (MAPK) p38 signaling [9].

Senescence has also been described as a physiological mechanism in healthy tissues, contributing to tissue regeneration and playing a pivotal role during embryogenesis [10]. Senescent cells are produced throughout life and play beneficial roles in a variety of processes besides embryogenesis, including wound healing, host immunity, and tumor suppression [11]. This beneficial dimension of senescence complements its better-known pathological roles and should be integrated into a comprehensive understanding of its function across the lifespan.

Our current comprehension of cellular senescence traces back to the pioneering work of Leonard Hayflick and Paul Moorhead in the 1960s. Their experiments revealed that normal human fibroblasts cultured in vitro have a limited capacity for cell division [12]. This limitation was subsequently linked to telomere shortening—a gradual loss of repetitive TTAGGG sequences at chromosomal ends during replication—which ultimately compromises genome stability [13]. This specific type of growth arrest is now recognized as replicative senescence [14].

Cellular senescence refers to a durable and typically irreversible halt in cell proliferation that occurs in otherwise dividing cells [15]. This state can be triggered by both internal and external stressors, such as DNA damage, oxidative conditions, mitochondrial impairment, or oncogenic activation [16,17]. A defining feature of senescent cells is the development of a senescence-associated secretory phenotype (SASP), a complex, pro-inflammatory secretome comprising cytokines, chemokines, proteases, and growth factors [18]. In addition to the SASP, senescent cells display altered metabolism, macromolecular damage, chromatin remodeling, and resistance to apoptosis [19].

Notably, senescence does not represent a single, uniform process. Instead, it encompasses a broad and diverse set of cellular responses, which can differ based on cell type, tissue environment, and the specific triggering factors. Over several decades of research, scientists have identified multiple senescence subtypes, each defined by unique initiating signals and biological consequences [20].

One of the most extensively studied senescence pathways is DNA damage-induced senescence, which occurs when cells encounter irreparable DNA lesions. These can arise from internal sources, like replication stress or telomere shortening, or from external exposures such as radiation and chemotherapeutic agents [21]. Another critical form is oncogene-induced senescence, initiated by abnormal oncogene activation or suppression of tumor-suppressing genes, functioning as an anti-cancer defense [10]. Additionally, therapy-induced senescence may result from anticancer interventions like chemotherapy or targeted therapies, originally thought to be beneficial but now also associated with resistance and tumor recurrence [22]. Regardless of the initiating factor, senescent cells typically share core traits such as SASP production, persistent cell-cycle arrest, chromatin remodeling, and distinct differences from other states like apoptosis or quiescence.

A growing body of evidence from animal models has established a causal role for senescent cells and the SASP in aging-related pathologies. In mice, the experimental clearance of senescent cells delays the onset or severity of multiple conditions—including sarcopenia, osteoarthritis, atherosclerosis, and cognitive decline—while improving tissue homeostasis and extending lifespan [23]. These findings have sparked considerable interest in senotherapeutics, including senolytic agents that eliminate senescent cells and senomorphic compounds that modulate the SASP [24,25]. However, the successful clinical translation of such approaches requires a better understanding of senescence and SASP biology in humans [26].

Due to its overlap with common inflammatory processes, distinguishing cellular senescence from inflammation can be challenging. Many SASP factors like MCP-1, TNF-α, and a number of interleukins are also produced during infections, wound healing, and autoimmune diseases [27]. The identification of specific markers or signatures of the SASP that are distinct from those of general inflammation is of great significance. Researching multi-marker signatures, such as persistent co-expression of pro-inflammatory cytokines (IL-6, IL-8) with extracellular matrix modulators (MMPs, TIMPs) and growth regulators (IGFBPs), could be proven useful in chronic diseases characterized by persistent inflammation that also promote cellular senescence.

To date, relatively few human studies have directly evaluated the role of SASP proteins in aging-related traits. Some studies have reported associations between a subset of circulating SASP proteins and specific health outcomes, such as reproductive aging [28], surgical recovery [29], physical performance [30], and depressive symptoms in late life [31]. Other proteomic investigations have found that specific SASP proteins have strong associations with chronological aging, chronic disease, and mortality [32]. Combining previously collected data from two independent, population-based cohorts, a study identified SASP proteins that are not only associated with chronological age, but also correlated with specific functional and physiological traits linked to aging [33].

Given its complexity and heterogeneity, measuring the SASP requires multiple methodological approaches that capture molecular regulation at transcriptional, translational, and functional levels across various biological sources. This presents a significant challenge for the field, particularly given the limitations of current detection methodologies. Commonly used biomarkers of senescence are often nonspecific, variably expressed, and inconsistently reliable, leading to considerable ambiguity in identifying senescence in both experimental and clinical settings. A recent meta-review highlighted this issue, demonstrating that correlations between senescence and aging-related tissue changes differed substantially depending on the biomarkers and detection techniques employed [34].

## 2. Methods of Measuring SASP

This review outlines the main techniques that have been used to date in order to quantify the SASP, categorized by molecular measurement level—RNA, protein, or activity—and sample source—cell culture, tissues, or systemic fluids. Table 1 describes SASP measurement methods categorized by level, technique, and sample type.

### 2.1. RNA-Level Analyses

#### 2.1.1. Quantitative Reverse Transcription PCR (qRT-PCR)

Quantitative reverse transcription PCR (qRT-PCR) is one of the most widely used molecular biology techniques to measure gene expression. qRT-PCR involves two primary steps: the reverse transcription of RNA into complementary DNA (cDNA) using reverse transcriptase, followed by amplification of specific gene targets using polymerase chain reaction with fluorescent detection. The real-time monitoring of fluorescence, typically via SYBR Green or TaqMan probes, allows quantification of transcript abundance in relative or absolute terms [52]. In the context of senescence research, it is particularly valuable for quantifying the transcriptional activation of SASP components such as pro-inflammatory cytokines, chemokines, growth factors, and matrix remodeling enzymes. The technique offers high sensitivity, specificity, and quantitative accuracy, making it ideal for studying SASP gene dynamics in vitro and in vivo settings.

In senescence studies, qRT-PCR has been instrumental in establishing the SASP as a defining feature of various senescence models. For example, Kuilman et al. used qRT-PCR to demonstrate increased mRNA levels of IL6 and IL8 in human fibroblasts undergoing oncogene-induced senescence [35]. Similarly, Rodier et al. utilized qRT-PCR to link persistent DNA damage signaling to sustained expression of IL-6, IL-1A, and other SASP factors [53].

qRT-PCR is also employed to monitor changes in SASP gene expression following pharmacological or genetic interventions. For instance, Laberge et al. used this method to show that inhibition of the mTOR pathway via rapamycin leads to a significant downregulation of IL-1A, IL-6, and other SASP-related transcripts, linking translational control to SASP modulation [36]. qRT-PCR is particularly advantageous when analyzing samples with limited RNA quantity, such as sorted cell populations or small tissue biopsies. The use of specific primers allows targeted quantification of key SASP components, making it a reliable tool for senescence diagnostics. Moreover, advancements in high-throughput qRT-PCR platforms now allow for simultaneous measurement of dozens to hundreds of SASP-related genes across large sample cohorts, facilitating comparative and longitudinal studies [54].

Despite its utility, qRT-PCR has limitations. It measures transcript levels, which may not directly correlate with protein abundance due to post-transcriptional regulation. Therefore, discrepancies between mRNA and protein levels may be observed in certain senescent cell models. The SASP, in its earliest characterization, encompassed roughly fifty biomolecules—including cytokines, chemokines (CXCLs), growth factors, and proteolytic enzymes. These were predominantly identified using biased methodologies such as antibody-based arrays or targeted transcriptional profiling approaches. Although such techniques have significantly advanced our understanding of the senescent cell phenotype, they inherently provide a partial view. This is where proteomic analysis emerges as a powerful counterpart.

An alternative that could offer methodological advantages to qPCR is digital PCR (dPCR). By partitioning a sample into thousands to millions of microreactions, dPCR enables absolute quantification of nucleic acid targets without the need for standard curves, thereby eliminating dependence on amplification efficiency assumptions. This partitioning also enhances sensitivity for detecting rare targets and increases tolerance to PCR inhibitors, as their effects are diluted among partitions. Furthermore, dPCR provides superior precision and reproducibility at low template copy numbers, making it particularly suitable for applications requiring high analytical accuracy. However, dPCR also has notable limitations. Its dynamic range is narrower than that of qPCR, as high template concentrations can saturate partitions, necessitating prior dilution. Throughput is generally lower, and assay times can be longer due to additional partitioning and detection steps. Moreover, the technique requires specialized instrumentation and consumables, leading to higher per-sample costs [55,56].

#### 2.1.2. RNA Sequencing (RNA-Seq)

RNA sequencing (RNA-seq) has emerged as a transformative technology in transcriptomic analysis, enabling unbiased, high-throughput quantification of gene expression across the entire transcriptome. It involves the extraction of total or polyadenylated RNA, followed by fragmentation, reverse transcription into cDNA, library preparation, and high-throughput sequencing. The resulting sequences are aligned to a reference genome or transcriptome to quantify gene expression levels [57].

In senescence research, RNA-seq has an important role for dissecting the complexity and heterogeneity of the SASP, especially in contexts where its composition is variable or unknown. Unlike targeted approaches such as qRT-PCR, RNA-seq does not rely on prior knowledge of gene sequences, thus offering a comprehensive view of transcriptional changes that occur during cellular senescence. This approach allows not only the detection of differentially expressed genes but also the discovery of novel transcripts, splice variants, and non-coding RNAs that may contribute to SASP regulation.

A landmark example of RNA-seq application in SASP profiling comes from Basisty et al., who generated a detailed “SASP Atlas” by performing RNA-seq and proteomics across multiple human cell types, including fibroblasts, endothelial cells, and preadipocytes, undergoing various senescence triggers (e.g., ionizing radiation, replicative exhaustion, oncogene activation). The study revealed both core SASP factors (e.g., IL-6, IL-8, MMP-1) and stimulus-specific components, underscoring the heterogeneity of the SASP and the importance of cell type and senescence inducer in shaping secretory profiles [58].

Other studies have leveraged RNA-seq to identify SASP expression patterns in specific tissues or disease models. For example, Casella et al. used RNA-seq to compare senescence profiles of in human fibroblasts, human umbilical vein endothelial cells, and human alveolar endothelial cells through different triggers of senescence [37]. RNA-seq is also instrumental in identifying transcriptional regulators of the SASP. In Herranz et al., RNA-seq was applied to showcase that mTOR controls the SASP by regulating the translation of the MK2/MAPKAPK2 kinase through 4EBP1 [38]. Similarly, Hoare et al. used RNA-seq to study the effect of NOTCH1 activity on SASP through two distinct secretomes, one representing TGF-β and the other pro-inflammatory cytokines [59].

Single-cell RNA sequencing (scRNA-seq) can offer advantages over bulk RNA sequencing for the analysis of tissues affected by cellular senescence. Bulk RNA-seq measures average transcript abundance across all cells, thereby obscuring heterogeneity and potentially masking transcriptional profiles of rare senescent cells. In contrast, scRNA-seq enables the identification and characterization of individual senescent cells within complex tissues, revealing subpopulations that differ by senescence trigger, stage, or cell type. This resolution facilitates the detection of co-expression patterns involving canonical senescence markers, alongside SASP factors. Furthermore, integration of scRNA-seq with spatial transcriptomics can localize senescent cells within tissue architecture, while trajectory inference can elucidate transcriptional transitions toward senescence [60,61]. Limitations of its use could be higher cost, greater technical complexity, and reduced sensitivity for low-abundance transcripts.

A human aging study employed scRNA-seq data to characterize SASP features in the aging testis, identifying IGFBP7—expressed in Leydig cells—as a novel SASP-associated gene and potential biomarker of testicular aging [39]. Voight et al. employed scRNA-seq to characterize transcriptional diversity among senescent cells, showing that SASP expression varies markedly between individual cells even within a clonal population [62]. This variability may explain differences in SASP function in vivo and suggests that cellular context plays a major role in modulating secretory behavior.

Despite its strengths, RNA-seq has its limitations in SASP research. Transcript levels do not always correlate with protein abundance or activity due to post-transcriptional, translational, or secretion-level regulation. Moreover, the data complexity of RNA-seq requires robust bioinformatic pipelines for quality control, alignment, normalization, and statistical analysis. Tools such as DESeq2 and edgeR are commonly used for differential expression analysis [63]. When used in combination with complementary proteomic and functional analyses, they help create a multi-dimensional profile of the SASP that reflects both cellular state and biological impact.

#### 2.1.3. RNA in Situ Hybridization

RNA in situ hybridization (RNA-ISH) is a molecular technique used to detect specific RNA molecules (e.g., mRNAs, long non-coding RNAs, viral RNAs) directly within intact cells and tissues, preserving spatial context. It allows researchers to identify where a transcript is expressed (e.g., which cell types or tissue regions) and how much is being expressed at the level of individual cells. Synthetic RNA or DNA probes are designed to be complementary to a specific RNA sequence, and the labeled probe binds to the target RNA within fixed cells or tissue sections. The hybridized probes are visualized using chromogenic methods (color development using alkaline phosphatase or HRP) or fluorescent labels (multiplexed RNA-ISH, e.g., RNAscope, ViewRNA) [64,65].

Lorda-Diez et al. used RNA-ISH while studying cell senescence and DNA damage in the remodeling processes accounting for heart morphogenesis. They found intensified expression of secreted factors characteristic of the senescent secretome, such as pro-inflammatory cytokines (IL-1β, IL-6), growth factors (IGF1, IGFBP5, TGF-βs), and tissue remodeling factors (ADAMTS9, MMP-2, MMP-9) [66].

RNA-ISH also provides the ability to study novel non-coding RNAs. Recent studies have begun to uncover the role of long non-coding RNAs (lncRNAs) and circular RNAs (circRNAs) in the regulation of the SASP. For example, Lin et al. used RNA-ISH to identify lncRNA MALAT1 in gallbladder cancer cells, with their results suggesting that it potentiates growth and inhibits senescence by antagonizing ABI3BP in these cancer cells [67].

Thus, RNA in situ hybridization is a powerful, spatially resolved method for identifying senescent cells and their secretory phenotypes within tissues. It complements bulk RNA analysis and can link SASP gene expression to histological features—an especially valuable feature for aging, cancer, and degenerative disease research. Of course it has its limitations as it requires fixed tissue. In addition, there are difficulties in identifying targets that have low RNA copies, and it has lower throughput compared to transcriptomics [68].

### 2.2. Protein-Level Analyses

#### 2.2.1. Cell Culture Supernatants

Enzyme-Linked Immunosorbent Assay (ELISA)

Enzyme-linked immunosorbent assay (ELISA) is a widely employed immunological technique used to quantitatively measure specific proteins in complex biological samples. ELISA operates on the principle of antibody-antigen specificity. A capture antibody is immobilized on a solid surface (typically a 96-well microplate), which selectively binds the target protein in the sample. A secondary detection antibody, conjugated to an enzyme such as horseradish peroxidase (HRP), binds to the captured antigen, forming a “sandwich” complex. Upon addition of a substrate (e.g., TMB), a measurable colorimetric change is produced, which is directly proportional to the concentration of the target analyte. The reaction is then quantified using a plate reader, with high sensitivity and reproducibility [40,69].

In the context of cellular senescence research, ELISA is a fundamental tool for detecting and quantifying soluble SASP components—cytokines, chemokines, growth factors, and matrix remodeling enzymes—in conditioned media, tissue lysates, plasma, and other bodily fluids. For instance, Coppé et al. utilized ELISA to measure elevated levels of IL-6 and IL-8 in the conditioned media of human fibroblasts undergoing oncogene-induced senescence (OIS) [70]. These findings complemented transcriptomic data and provided direct evidence of increased secretion of SASP factors. Similarly, Acosta et al. demonstrated via ELISA that IL-1α functions upstream of the IL-6/IL-8 axis in the SASP regulatory cascade, highlighting the assay’s utility in dissecting cytokine hierarchies [71]. In the study by Wiley et al., it was used in conjunction with TMT-based MS to quantify secreted proteins from senescent and proliferating fibroblasts, revealing distinct proteomic signatures associated with different senescence triggers. Notably, the researchers discovered that while some proteins were consistently secreted regardless of senescence inducer (e.g., IL-8, MMP-1), others were unique to specific senescence pathways, illustrating the pathway-dependent nature of the SASP [72].

ELISA remains a gold-standard technique for the quantitative analysis of soluble SASP components, particularly in conditioned media and body fluids. Its simplicity, specificity, adaptability, and high sensitivity (typically in the picogram per milliliter range) make it indispensable for both mechanistic and translational studies of cellular senescence. However, it does have its limitations. It is a targeted assay, requiring prior knowledge of the proteins of interest and the availability of high-affinity, specific antibodies. Cross-reactivity and batch-to-batch variability in antibody performance can affect assay reliability. Moreover, ELISA cannot detect unknown or unexpected components of the SASP, nor does it provide information about post-translational modifications, which can modulate the function or activity of SASP proteins. Thus, it is most effective when used for quantitative validation of candidate SASP factors initially identified by unbiased transcriptomic or proteomic screening. When integrated with complementary approaches such as RNA-seq, qRT-PCR, and mass spectrometry, ELISA contributes to a robust, multi-level characterization of the SASP, facilitating both basic research and the development of senescence-targeted interventions [73,74].

Western Blotting

Western blotting is a classical and widely used protein detection technique that plays a critical role in senescence research by enabling the qualitative and semi-quantitative assessment of individual SASP components. While not as high-throughput as ELISA or mass spectrometry, Western blotting offers key advantages in specificity, validation of protein identity, and the detection of post-translational modifications (PTMs), making it useful for characterizing SASP regulators and effectors [41]. The technique involves the separation of often denatured proteins by molecular weight through SDS–PAGE (sodium dodecyl sulfate–polyacrylamide gel electrophoresis), followed by transfer to a membrane (typically PVDF or nitrocellulose) and probing with specific primary and secondary antibodies. Detection is typically achieved through chemiluminescence or fluorescence, providing visual confirmation of protein presence and relative abundance [42,75].

In the context of SASP, Western blotting is commonly used to validate the presence of secreted or intracellular regulators such as IL-6, IL-1α, TNF-α, matrix metalloproteinases (e.g., MMP-1, MMP-3), and signaling proteins involved in SASP induction pathways, including components of the NF-κB, mTOR, and p38 MAPK signaling axes. For instance, Orjalo et al. used Western blotting to demonstrate that membrane-bound IL-1α, a key upstream regulator of SASP gene expression, is expressed at higher levels in senescent fibroblasts than in proliferating controls [76]. This study provided mechanistic insight into how IL-1α activates NF-κB signaling and perpetuates the SASP. Similarly, Herranz et al. used Western blotting to confirm changes in the phosphorylation state of mTOR in therapy-induced senescence, supporting a regulatory role of mTOR signaling in SASP protein production [38].

Western blotting is also used to monitor the intracellular accumulation of SASP-related transcription factors and modulators. For example, Rodier et al. demonstrated persistent DNA damage signaling in senescent cells by Western blot detection of ATM, NBS1, and CHK2, which are associated with the chronic DDR (DNA damage response) that sustains SASP gene expression [53]. Moreover, Western blotting is capable of detecting post-translational modifications—such as phosphorylation, acetylation, and ubiquitination—that critically regulate SASP protein activity and stability [77].

While Western blotting is often performed on cell lysates, it can also be applied to concentrated conditioned media to detect secreted SASP proteins. However, this requires protein concentration steps (e.g., ultrafiltration or TCA precipitation) and careful normalization due to variable protein yields in secretomes. Some studies, including Acosta et al., have used Western blotting to detect secreted IL-6 and IL-8 in media from senescent cells, thereby validating qRT-PCR or RNA-seq findings at the protein level [71].

Despite its strengths, Western blotting has notable limitations, since it is less sensitive than ELISA for detecting low-abundance secreted proteins and requires high-quality, validated antibodies to ensure specificity. Quantification is semi-quantitative at best, as signal intensity can be affected by factors such as antibody affinity, exposure time, and transfer efficiency. Moreover, the method is relatively low-throughput and labor-intensive, limiting its utility for large-scale screening of SASP components [78,79].

Mass Spectrometry

Mass spectrometry (MS) has emerged as a powerful and unbiased approach for the comprehensive profiling of the SASP at the protein level. Unlike targeted methods such as ELISA or Western blotting, MS-based proteomics allows for the simultaneous identification and quantification of hundreds to thousands of proteins in complex biological samples without prior assumptions. This makes it uniquely suited for uncovering the full diversity and dynamics of the SASP across different cell types, senescence triggers, and biological contexts [80]. The general workflow of mass spectrometry-based proteomics involves the extraction and digestion of proteins (commonly using trypsin) from samples such as cell culture-conditioned media, tissue interstitial fluid, or blood plasma. The resulting peptides are separated by liquid chromatography (LC) and then analyzed using tandem MS, where their mass to charge ratios are measured and sequenced to infer protein identity and abundance [43,81].

A landmark study by Basisty et al. exemplifies the application of MS in SASP research. The authors used data-independent acquisition (DIA)-based MS to systematically analyze the secretomes of multiple human cell types (fibroblasts, epithelial cells, endothelial cells, and preadipocytes) undergoing senescence induced by irradiation, oncogene activation, or replicative exhaustion. Their work produced the first comprehensive “SASP Atlas,” which cataloged both core and context-specific SASP proteins—including cytokines (e.g., IL-6, CXCL1), extracellular matrix components (e.g., fibronectin, SERPINs), proteases (e.g., MMPs, cathepsins), and unexpected mediators like growth differentiation factor 15 (GDF15) and stanniocalcin 1 (STC1) [58]. These findings expanded the functional landscape of the SASP and highlighted its complexity beyond traditional inflammatory markers.

Samsonraj et al. used MS-based proteomics to analyze conditioned media from replicatively senescent bone marrow-derived human mesenchymal stem cells (MSCs), comparing them to early-passage controls. They identified secreted proteins uniquely elevated in senescent MSCs, including IL-6, MMP-1, SERPINE1, OPG, and COL1A1. These findings were validated under oxidative and irradiative stress, and select proteins (e.g., IL-6, OPG) were also increased in plasma of aged rats [44]. Özcan et al. conducted an unbiased LC–MS/MS (liquid chromatography–tandem mass spectrometry) analysis of secretomes from bone marrow and adipose mesenchymal stromal cells which were exposed to oxidative stress, irradiation, doxorubicin, and replicative exhaustion. They identified 11 proteins shared across cells subjected to senescence triggers and three key signaling pathways that could suggest a conserved core SASP signature [82].

One of the key strengths of MS in SASP research is its ability to detect post-translationally modified proteins, alternative splicing products, and proteolytically cleaved fragments that are often missed by transcriptomics or antibody-based methods [46,83]. For instance, matrix metalloproteinases (MMPs), a class of enzymes often enriched in the SASP, are secreted in latent forms and activated extracellularly—processes that are captured effectively by MS analysis of conditioned media but not by RNA-seq or standard Western blotting [47]. Moreover, MS is particularly well-suited for biomarker discovery in clinical samples [84].

Nevertheless, MS-based proteomics comes with challenges. The technique requires specialized instrumentation, expert data analysis, and careful sample preparation to minimize artifacts and improve detection sensitivity. Low-abundance cytokines and chemokines may fall below detection thresholds unless samples are fractionated or enriched. Additionally, the complexity and dynamic range of biological fluids like plasma or serum necessitate depletion of abundant proteins (e.g., albumin) to enhance SASP protein recovery [85].

Multiplex Assays (Luminex, MSD)

Multiplex immunoassays have emerged as highly valuable tools for the quantitative measurement of multiple protein analytes simultaneously. They provide distinct advantages over conventional single-analyte ELISAs for the detection of SASP components, particularly when the goal is to characterize complex secretory signatures. By enabling the simultaneous quantification of multiple cytokines, chemokines, growth factors, and proteases within a single sample, multiplex platforms allow comprehensive profiling of the diverse SASP repertoire, which typically includes IL-6, IL-8, MCP-1, matrix metalloproteinases, and insulin-like growth factor binding proteins. This parallel detection reduces the sample volume required, an important consideration when working with limited material from primary senescent cultures or small tissue biopsies. Multiplex assays also improve experimental efficiency by reducing assay time and labor compared with running multiple ELISAs. Furthermore, the ability to obtain relative abundance patterns for multiple factors from the same sample facilitates identification of core and context-specific SASP signatures. Although initial costs may be higher, multiplex approaches can offer greater overall cost-effectiveness for broad SASP profiling [86].

Two of the most widely adopted platforms for multiplexing are Luminex xMAP technology and Meso Scale Discovery (MSD) electrochemiluminescence (ECL). Both systems offer high sensitivity, broad dynamic range, and the ability to interrogate dozens of secreted factors in parallel—attributes that are particularly advantageous when profiling the SASP in tissue-derived samples, where sample quantity and composition can be limiting [87].

Luminex Assays

Luminex technology employs microsphere-based flow cytometry. Each microsphere bead is dyed with a unique fluorescent signature and coated with a capture antibody specific to a target protein. Upon incubation with a tissue lysate, the analyte binds its corresponding bead. A biotinylated detection antibody and a streptavidin-phycoerythrin conjugate are then added, and the bead-associated signal is quantified by flow-based detection. This format is particularly useful in SASP studies involving tissue homogenates, allowing researchers to simultaneously measure inflammatory cytokines (e.g., IL-6, IL-8, TNF-α), chemokines (e.g., CCL2/MCP-1, CXCL1), and matrix remodeling proteins (e.g., MMP-3, TIMPs) [45].

In early-senescent mesenchymal stromal cells (MSCs), researchers compared conditioned media from young and aged donors using an R&D Systems Luminex panel. They measured a customized SASP set (IL-1α, IL-8, IL-6, MCP-1, IL-1β, TNF-α, GRO-β, and CCL4) and also applied assays to bone marrow sera, confirming age-associated increases in these SASP cytokines [88]. Luminex assays have also been used to assess changes in SASP composition in response to therapeutic interventions. A study by Abdelgawad et al. investigated the effects of metformin on doxorubicin-induced endothelial senescence. Using Luminex assays, researchers measured the secretion levels of SASP factors such as IL-6, TNF-α, CXCL1, CXCL2, MCP-1, MCP-3, MIP-1α, and MMP-3 in the culture supernatants of senescent endothelial cells [48]. The study found that metformin treatment significantly reduced the secretion of these pro-inflammatory cytokines and chemokines, suggesting its potential in mitigating vascular inflammation associated with cellular senescence. Camell et al. used a Luminex assay to evaluate how exposure to pathogen-associated molecular pattern (PAMP) factors and treatment with senolytic drugs (e.g., fisetin, dasatinib, and quercetin) modulates the circulating SASP proteome in aged mice. The study revealed significant reductions in pro-inflammatory and pro-fibrotic SASP components, supporting the utility of proteomics in evaluating treatment efficacy and identifying biomarkers of senescent cell clearance [89].

Meso Scale Discovery (MSD) Assays

MSD technology utilizes multi-spot electrochemiluminescent detection on patterned plates, where each spot is coated with a different capture antibody. Upon sample incubation and binding of target analytes, detection antibodies labeled with electrochemiluminescent tags (e.g., ruthenium complexes) are introduced. When voltage is applied, light is emitted from each spot in proportion to analyte concentration, enabling high-sensitivity multiplex detection. MSD is particularly valued for its low background signal, broad dynamic range (up to 5–6 logs), and excellent reproducibility [90]. It has been widely used in both preclinical and clinical SASP studies.

For example, Alimbetov et al. investigated the effects of small molecule inhibitors targeting p38 MAPK and MK2 on the SASP in human fibroblasts. Conditioned media from senescent cells were analyzed using MSD multiplex immunoassays to measure cytokines such as GM-CSF, IL-1β, IL-6, IL-8, IL-10, TNF-α, and IFN-γ. The study demonstrated that these inhibitors effectively reduced the secretion of pro-inflammatory SASP factors [91]. A study by Smith et al. developed a model to examine molecular mechanisms of macrophage aging. Conditioned media from macrophages at various time points were collected and analyzed using MSD U-PLEX assays. The assays quantified levels of IL-6, KC-GRO, MCP-1, MMP-9, TNF-α, IL-1β, and IL-10, providing insights into the dynamic changes in SASP components during macrophage aging [92]. Alessio et al. studied the progression of senescence in mesenchymal stromal cells (MSCs) induced by ionizing radiation. Conditioned media from early and late senescent MSCs were analyzed using MSD assays to profile SASP components. The study revealed temporal changes in the SASP composition, highlighting differences between early and late senescent states [93].

Luminex and MSD multiplex immunoassays provide powerful, scalable platforms for the quantitative analysis of SASP components in cell culture supernatants samples. Their ability to measure dozens of analytes simultaneously from small sample volumes makes them ideal for in vivo studies of tissue aging, senescence burden, and response to senescence-targeted therapies. By enabling direct comparison of SASP expression across tissue types, conditions, and species, these assays serve as indispensable tools in both basic and translational senescence research [94]. Of course these assays have also their limitations. Cross-reactivity and varying affinity of antibodies can introduce quantification errors with validation of antibody pairs being essential. Moreover, while they allow detection of known SASP proteins, they are inherently targeted platforms and cannot detect novel or unexpected secreted factors [95]. For discovery purposes, integration with untargeted mass spectrometry remains valuable.

#### 2.2.2. Plasma and Serum

The detection of circulating SASP components in plasma and serum has become an increasingly important area of research, particularly in the context of aging, age-related diseases and the development of senescence-targeted therapeutics. Unlike cell culture or tissue-based studies, plasma and serum analyses provide a minimally invasive way to assess systemic senescence burden and hold potential for biomarker discovery, longitudinal monitoring, and clinical translation. Circulating SASP components are typically measured in plasma (obtained using anticoagulants like EDTA or heparin) or serum (obtained after clotting), with minor differences in protein content due to clotting-related release of platelet factors. The circulating SASP includes a diverse array of secreted proteins which are detectable in circulation, where they may contribute to systemic inflammation, tissue dysfunction, or inter-organ communication during aging and disease [29,96].

Detection Methods

A variety of techniques are used to measure SASP components in plasma and serum. Multiplex immunoassays (Luminex, MSD) offer high-throughput measurement of dozens of proteins. Picca et al. utilized the Bio-Plex Pro Human Cytokine 27-plex Assay (Luminex xMAP technology) and the ELLA™ automated immunoassay system to identify elevated levels of inflammatory and senescence-related markers (IL-1β, IL-6, TNF-α, activin A, FGF21, GDF15, GFAP, ICAM-1, SERPINE1, and TIMP-1) in older adults with physical frailty and sarcopenia, suggesting a link between circulating SASP factors and age-related physical decline [97]. Results from LIFE Study concerning SASP markers and mild cognitive impairment found associations between higher plasma levels of certain SASP markers and increased odds of mild cognitive impairment and dementia over a 24-month period. Using Luminex xMAP multiplex magnetic bead-based immunoassays analyzed on the MAGPIX System, they assessed 27 SASP biomarkers, including MPO, MMP-1, and MMP-7 [98]. Manna et al. demonstrated heterogeneous differences in SASP profiles in maternal plasma samples from cases of pre-eclampsia and intrauterine growth restriction (IUGR), indicating potential biomarkers for these conditions measuring IL-6, IL-8, IL-13, IL-1α, IFN-γ, MCP-1, MIP-1α, and MMP-3. They utilized the Human U-Plex kit and MMP-3 Ultra-sensitive kit from MSD [49].

ELISA is highly sensitive for individual factors. In many studies, it was combined with multiplex immunoassays to measure specific SASP components. In a study by Schafer et al., ELISAs were used to quantify plasma levels of SASP proteins activin A and plasminogen activator inhibitor 2 (PAI-2) in order to find a correlation between their levels and chronological age, frailty, and age-related pathologies [29]. Wang et al. measured the plasma levels of circulating GDF15 as potential biomarker of metabolic dysregulation and aging in people living with HIV [99].

The ability to detect SASP factors in plasma and serum provides a foundation for the development of senescence biomarkers that could be used in aging and frailty assessments, prediction of chronic disease risk (e.g., cardiovascular disease, osteoarthritis, neurodegeneration), monitoring of senescence-modifying interventions (e.g., senolytics, caloric restriction, exercise), and stratification of patients for clinical trials [93, 94]. The analysis of circulating SASP components in plasma and serum provides a crucial bridge between experimental senescence research and clinical application [51]. The integration of machine learning and systems biology approaches with large-scale proteomic and clinical datasets is expected to further refine SASP signatures and improve their specificity for senescence [50]. Additionally, longitudinal studies tracking SASP factors in plasma/serum across lifespan or in response to therapies will be key for establishing robust, clinically meaningful biomarkers.

Despite its promise, the detection of circulating SASP components poses several challenges. SASP factors secreted by senescent cells may be diluted in the bloodstream, making them harder to detect, particularly for proteins with short half-lives. Circulating proteins may arise from multiple cell types, not exclusively senescent cells, necessitating complementary methods to confirm senescence origin. Moreover, many SASP proteins overlap with general inflammatory markers, complicating the interpretation in settings of infection, injury, or other comorbidities [100,101]. Lastly, collection, processing, and storage of plasma/serum samples can influence protein integrity and reproducibility.

Protein Localization

Understanding the localization of SASP proteins is critical for elucidating their functional roles, cellular origins, and tissue-level impacts in the context of cellular senescence. While bulk measurements (e.g., ELISA, mass spectrometry) can quantify the abundance of SASP components in culture media, tissue lysates, or circulation, they often lack spatial resolution. Protein localization techniques enable researchers to determine where within tissues or cells SASP factors are produced, stored, or act, providing crucial insights into senescence biology and its systemic effects. SASP molecules function not only in autocrine or paracrine signaling but also diffuse into surrounding tissues and circulation [102]. Therefore, establishing precise spatial relationships, such as whether SASP proteins are localized to senescent cells themselves, neighboring immune cells, or extracellular compartments, is fundamental to understanding their mechanisms of action [103]. Several well-established and emerging techniques are employed to visualize and localize SASP proteins in situ, typically within tissues or cell populations [104].

(i) Immunohistochemistry

Immunohistochemistry (IHC) uses antibody-based detection of proteins in formalin-fixed, paraffin-embedded (FFPE) or frozen tissue sections. A chromogenic or fluorescent label is used to visualize the antibody–protein complex. Studies demonstrate the utility of IHC in localizing and quantifying SASP components within human tissues, providing insights into the role of cellular senescence in various pathological conditions [105,106]. Vital et al. assessed the involvement of SASP factors in benign prostatic hyperplasia (BPH), using IHC analysis to detect the expression of cathepsin D and IL-1α in BPH tissues. Multifocal epithelial expression of both were observed, suggesting a role for SASP in the development of BPH [107].

(ii) Immunofluorescence

Immunofluorescence (IF) is a variant of IHC using fluorescently labeled antibodies. Fluorescently labeled antibodies bind to target proteins, and when excited by specific wavelengths of light, these fluorophores emit fluorescence that can be detected using fluorescence microscopy [108]. It is commonly used in both tissue sections and cultured cells. Xu et al. created a reversible immortalized cell line by expressing SV40T in human keratinocytes. Along with other methods they used IF to detect SASP-associated proteins MMP-1, SERPINB2, and VEGF-A after inducing a senescent phenotype by terminating SV40T expression [109]. Ruhland et al. used immunofluorescence staining to detect increased expression of IL-6 in stromal fibroblasts of mouse skin grafts when senescence was present [110].

## 3. Conclusions

The SASP is a complex and dynamic phenotype requiring integrated, multi-level measurement strategies. RNA-level techniques (qRT-PCR, RNA-seq) provide mechanistic insights into SASP regulation. Protein-level techniques (ELISA, Western blot, mass spectrometry) offer quantitative and broad assessments of SASP output, while multiplex assays extend these analyses to tissues and fluids. Immunostaining localizes SASP expression in senescent cells. Together, these methods provide a comprehensive toolkit for dissecting the SASP across models and contexts, with implications for aging, cancer, tissue repair, and senescence-targeted therapies.

Despite advances in biomedical research, the precise role of cellular senescence in human tissues—both in normal physiological states and in disease—remains far from fully understood. A particularly critical gap lies in understanding how sublethal genomic damage, a feature often present in rare genetic disorders, may act as an independent trigger for senescence. Without clear distinctions between cellular aging processes and inflammatory responses caused by an underlying disease, researchers risk conflating two distinct biological phenomena. This lack of clarity hinders efforts to define the exact molecular or structural markers that could reliably identify senescence in human tissues.

Equally important is the unresolved question of whether senescent cells should invariably be eliminated or instead selectively modulated. Evidence suggests that, under certain circumstances, senescence may serve protective or reparative roles, making a blanket eradication strategy potentially counterproductive. Understanding the conditions under which senescence is beneficial versus harmful will be vital for guiding therapeutic approaches. Ultimately, deepening our insight into these mechanisms will enable the design of more precise interventions, ones that can either suppress harmful senescence to slow disease progression or harness its advantageous effects to promote tissue health and resilience.

## Figures and Tables

**Table 1:** SASP measurement methods categorized by level, technique, and sample type.

Level	Method	Sample Type	Key Applications/Examples
**RNA**	qRT-PCR	Cell culture, tissue	IL-6/IL-8 in senescent fibroblasts [31]; IL-1α reduction with rapamycin [33]
	RNA-seq	Cell culture, tissue	SASP Atlas [35]; diversity in fibroblasts, endothelial cells [36]
	RNA In Situ Hybridization	Tissue (fixed sections)	Spatial detection of IL-6, IL-1β, MMPs [37]; MALAT1 lncRNA [38]
**Protein**	ELISA	Cell culture, plasma, serum	IL-6, IL-8 in OIS fibroblasts [39]; PAI-2/activin A in plasma [24]
	Western Blotting	Cell culture, tissue lysate	IL-1α [40]; mTOR phosphorylation [36]; MMPs [32]
	Mass Spectrometry	Cell culture, plasma, serum	SASP Atlas; senescent MSCs [41]; conserved secretome [42]
	Luminex Multiplex Assays	Cell culture, tissue, plasma	Cytokines in MSCs [43], senescent ECs [44]; frailty plasma [45]
	MSD (Meso Scale Discovery)	Cell culture, tissue, plasma	MK2/p38 inhibition [46]; macrophage aging [47]; human frailty studies [48]
**Localization**	Immunohistochemistry (IHC)	Tissue sections (FFPE)	IL-1α, Cathepsin D in BPH [49]
	Immunofluorescence (IF)	Cells, tissues	IL-6 in stromal fibroblasts [50]; MMP-1, SERPINB2 in keratinocytes [51]
	In Situ Hybridization (RNAscope)	Tissue (mRNA localization)	IL-1β, IL-6, MMP-2, MMP-9 in developing heart [37]

## Data Availability

Research data are available upon request.
